# Evaluation of patient immunocompetence for immune checkpoint inhibitor therapy using the psoas muscle index: a retrospective cohort study

**DOI:** 10.3389/fonc.2025.1499650

**Published:** 2025-02-06

**Authors:** Toshiaki Tsurui, Kazuyuki Hamada, Emiko Mura, Risako Suzuki, Nana Iriguchi, Tomoyuki Ishiguro, Yuya Hirasawa, Ryotaro Ohkuma, Masahiro Shimokawa, Hirotsugu Ariizumi, Yutaro Kubota, Atsushi Horiike, Satoshi Wada, Kiyoshi Yoshimura, Mayumi Tsuji, Yuji Kiuchi, Takuya Tsunoda

**Affiliations:** ^1^ Division of Medical Pharmacology, Showa University Graduate School of Medicine, Tokyo, Japan; ^2^ Department of Clinical Immuno-Oncology, Clinical Research Institute for Clinical Pharmacology and Therapeutics, Showa University, Tokyo, Japan; ^3^ Division of Medical Oncology, Department of Medicine, Showa University School of Medicine, Tokyo, Japan; ^4^ Pharmacological Research Center, Showa University, Tokyo, Japan; ^5^ Department of Chest Surgery, School of Medicine, Fukushima Medical University, Fukushima, Japan; ^6^ Department of Medical Oncology, Showa University Graduate School of Medicine, Tokyo, Japan; ^7^ Department of Clinical Diagnostic Oncology, Clinical Research Institute of Clinical Pharmacology and Therapeutics, Showa University, Tokyo, Japan

**Keywords:** immune checkpoint inhibitors, immunotherapy, lung neoplasms, sarcopenia, retrospective study

## Abstract

**Introduction:**

In patients with cancer, sarcopenia is an indicator of poor prognosis and is associated with an increased risk of chemotherapy-related adverse events. Skeletal muscle interacts with the immune system, and sarcopenia is associated with immune senescence. However, the association between sarcopenia and the response to immune checkpoint inhibitor (ICI) therapy remains unclear.

**Methods:**

This retrospective study included patients with advanced or recurrent non-small cell lung cancer treated with nivolumab or pembrolizumab monotherapy. The association between the psoas muscle index (PMI) and both clinical response and immune-related adverse events (irAEs) was assessed using logistic regression. The PMI was calculated as the cross-sectional area of the psoas muscle divided by the square of the height based on computed tomography scans performed before the initial administration of ICI therapy.

**Results:**

A total of 67 patients were included in the analysis. Logistic regression analysis showed that PMI was associated with the overall response (odds ratio [OR]: 1.52; 95% confidence interval [CI]: 1.04–2.22; *p* = 0.030) and the risk of severe irAEs (OR: 1.72; 95% CI: 1.05–2.80; *p* = 0.031).

**Conclusion:**

These findings suggest that PMI is both an indicator of prognosis and a surrogate marker of immunocompetence in predicting the clinical response to ICI therapy.

## 1 Introduction

Immune checkpoint inhibitors (ICIs) have demonstrated clinical benefits in patients with advanced cancer, including those with non-small cell lung cancer (NSCLC). Despite these advancements, the number of patients who benefit from ICIs remains limited, and no reliable predictive biomarkers have been identified. The mechanism of action of ICIs, which involves acting on the host immunity and stimulating the immune system against cancer cells, is fundamentally different from that of conventional cytotoxic chemotherapy. Hence, the effectiveness of ICIs is strongly dependent on patient immunocompetence, necessitating a comprehensive evaluation of patient immune status to predict responsiveness to ICI therapy.

Sarcopenia is defined as a loss of skeletal muscle mass and function. Its prevalence increases with age, and >40% of patients with advanced cancer are sarcopenic (1). In patients with cancer, sarcopenia is not only an independent factor associated with poor prognosis but is also associated with an increased incidence of adverse events caused by cytotoxic chemotherapy (2). Recent studies have demonstrated that skeletal muscle interacts with the immune system through various surface molecules and cytokines known as myokines, potentially linking sarcopenia to immune senescence (3, 4). Despite recent reports that sarcopenia is associated with a reduced clinical benefit of ICIs, it is unclear whether sarcopenia is merely a prognostic indicator or if it is associated with reduced effectiveness of ICI therapy through influencing the host immune system (5–11).

This study aimed to assess the relationship between skeletal muscle mass and the immune response to ICI therapy in patients with NSCLC. Skeletal muscle mass was evaluated using the psoas muscle index (PMI) measured on computed tomography (CT), a reliable indicator of skeletal muscle mass and sarcopenia status (12, 13).

## 2 Methods

### 2.1 Study design and data collection

This study had a retrospective cohort design. This study was designed as a retrospective cohort study. Patients diagnosed with advanced or recurrent non-small cell lung cancer (NSCLC) at our institution between 2016 and 2023 were included. The inclusion criteria consisted of patients with histologically confirmed advanced or recurrent NSCLC who received nivolumab or pembrolizumab monotherapy. Exclusion criteria included patients who did not have a pre-treatment abdominal CT scan within 4 weeks of initiating ICI therapy, as well as patients with poor-quality CT scans that were not suitable for PMI calculation, to minimize measurement errors. Baseline demographics, histology, prior chemotherapy, programmed cell death-ligand 1 (PD-L1) expression (evaluated using 28-8 or 22C3 pharmDx assays), serum albumin level, lymphocyte count, neutrophil-to-lymphocyte ratio, clinical outcomes, and adverse events were extracted from medical records. Many patients did not have an Eastern Cooperative Oncology Group Performance Status (PS) recorded; therefore, the PS was not included in the statistical analysis. Data collection was censored on May 1, 2023. This study was conducted in accordance with the principles of the Declaration of Helsinki. The Institutional Review Board, the Ethics Committee of Showa University School of Medicine, approved the study on March 3, 2023 (approval number: 22-058-A) and waived the requirement for informed consent because only medical records and images were reviewed.

### 2.2 Outcomes and sample size

The response to ICIs was evaluated using the Response Evaluation Criteria in Solid Tumors (version 1.1). Patients who showed complete response (CR) or partial response (PR) as the best overall response were defined as responders, and the others were defined as non-responders. Immune-related adverse events (irAEs) were diagnosed clinically, and the information was extracted from medical records. Severe irAEs were defined as any irAE that led to permanent discontinuation of ICIs. In the statistical analyses, age was considered as a potential confounding factor that influences both PMI and the outcomes. Using the “ten events per variable” rule and assuming an overall response rate (ORR) of 30% based on previous clinical trials, the minimal sample size was determined to be 60 in order to perform logistic regression analysis including PMI and age as variables (14–16). Sensitivity analyses were performed by substituting sex and albumin level for age as independent variables as they might influence both PMI and clinical outcomes. The Prognostic Nutritional Index (PNI) and Geriatric Nutritional Risk Index (GNRI) were also assessed as nutritional indices that may influence treatment outcomes. PNI was calculated using the formula: 10 × serum albumin level (g/dL) + 0.005 × total lymphocyte count (per mm³). GNRI was calculated using the formula: 14.89 × serum albumin level (g/dL) + 41.7 × current body weight/ideal body weight (17). These indices were analyzed in relation to the overall response rate (ORR), irAE incidence, progression-free survival (PFS), and overall survival (OS).

### 2.3 PMI

PMI was calculated as the cross-sectional area of the psoas muscle at the third lumbar vertebra divided by the square of the patient’s height. This method has been previously reported for evaluating sarcopenia in oncology patients (5–7). CT images used for PMI measurement were obtained before the initial administration of ICIs. The measurements were performed using SYNAPSE VINCENT software (Fujifilm Medical, Tokyo, Japan), which automatically identifies and measures the psoas muscle at the L3 level, minimizing the potential for operator error. For reference, we used established PMI cutoff values: 3.92 cm²/m² for female patients and 6.36 cm²/m² for male patients, as previously reported in healthy Asian populations without cancer (18). The study cohort was then categorized into “high-PMI” and “low-PMI” groups based on whether their PMI was above or below the median PMI, respectively, allowing for an unbiased division of the study population.

### 2.4 Statistical methods

Two-group comparisons were performed using the chi-square test and Student’s t-test for categorical and continuous variables, respectively. Multivariable logistic regression was used to assess the association between the overall response and the incidence of irAEs. Progression-free survival (PFS) and overall survival (OS) were calculated using the Kaplan–Meier method, and group differences were analyzed using the log-rank test. Cox regression was used to analyze the association between PMI and survival. There were no missing data on the variables used in the statistical analyses. All statistical analyses were performed using JMP version 17.0.0 (SAS Institute Inc., Cary, NC, USA) and R software version 4.1.0 (R Foundation for Statistical Computing, Vienna, Austria). Two-tailed *p* values < 0.05 were considered statistically significant.

## 3 Results

### 3.1 Baseline characteristics

Sixty-seven patients (52 male and 15 female) were identified and confirmed eligible for inclusion in the analysis. The baseline characteristics of the low- and high-PMI groups are summarized in **Table 1**. The patients’ mean age was 65.4 years, and their mean PMI was 4.62 cm^2^/m^2^. Both PMI and body mass index were significantly higher in the high-PMI group than in the low-PMI group (*p* < 0.001 and *p* = 0.002, respectively). PNI and GNRI were not different between two groups. No differences were observed in histopathological characteristics or the prevalence of liver or brain metastases based on the PMI status. Pembrolizumab was administered to patients in the high-PMI group more frequently than to those in the low-PMI group (41% vs. 15%; *p* = 0.018).

**Table 1:** Baseline characteristics of the patients stratified by PMI.

	High-PMI group (N = 34)	Low-PMI group (N = 33)	*p*
PMI (cm^2^/m^2^), mean (SD)	5.57 (1.24)	3.64 (0.88)	<0.001
BMI (kg/m^2^), mean (SD)	23.9 (4.77)	20.8 (3.15)	0.002
PNI, mean (SD)	44.2 (5.88)	42.0 (6.88)	0.173
GNRI, mean (SD)	98.2 (10.9)	94.2 (13.9)	0.188
Age (years), mean (SD)	63.7 (9.0)	67.1 (8.1)	0.105
Sex, n (%)			0.820
Female	8 (23.5%)	7 (21%)	
Male	26 (76.5%)	26 (79%)	
Histology, n (%)			0.441
Adenocarcinoma	24 (71%)	26 (79%)	
Squamous carcinoma	10 (29%)	7 (21%)	
Brain metastasis, n (%)			0.507
Yes	6 (18%)	8 (24%)	
No	28 (82%)	25 (76%)	
Liver metastasis, n (%)			0.144
Yes	6 (18%)	2 (6%)	
No	28 (82%)	31 (94%)	
PD-L1, n (%)			0.559
<1%	4 (12%)	6 (18%)	
1–49%	5 (15%)	7 (21%)	
≥50%	17 (50%)	11 (33%)	
Unknown	8 (23.5%)	9 (27%)	
Regimen, n (%)			0.018
Nivolumab	20 (59%)	28 (85%)	
Pembrolizumab	14 (41%)	5 (15%)	
Number of previous chemotherapy lines, n (%)			0.256
0	11 (32%)	5 (15%)	
1	13 (38%)	16 (48.5%)	
≥2	10 (29%)	12 (36%)	

BMI, body mass index; GNRI, Geriatric Nutritional Risk Index; PD-L1, programmed death-ligand 1; PMI, psoas muscle index; PNI, Prognostic Nutritional Index; SD, standard deviation.

### 3.2 PMI

**Figure 1:**
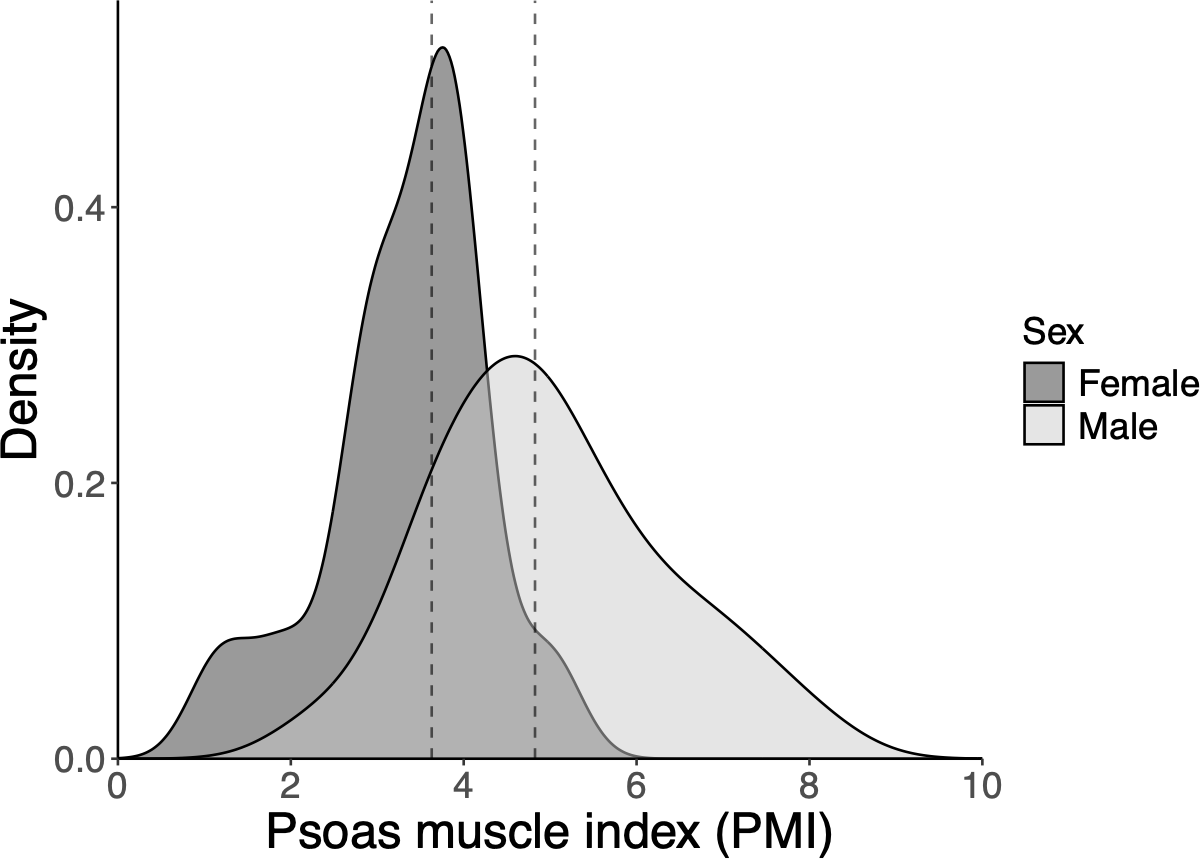
Density plot showing distribution of the psoas muscle index (PMI) according to sex. The dashed line represents the median PMI (females: 3.63 cm^2^/m^2^, and males: 4.825 cm^2^/m^2^). PMI, psoas muscle index.

The distribution of PMI in female and male patients is shown in **Figure 1**. The median PMI was 3.63 cm²/m² for female patients and 4.825 cm²/m² for male patients. Using the previously reported PMI cutoff values for healthy individuals without cancer (3.92 cm²/m² for female patients and 6.36 cm²/m² for male patients), 13 female patients (87%) and 43 male patients (83%) were classified as sarcopenic.

### 3.3 Treatment response

**Figure 2:**
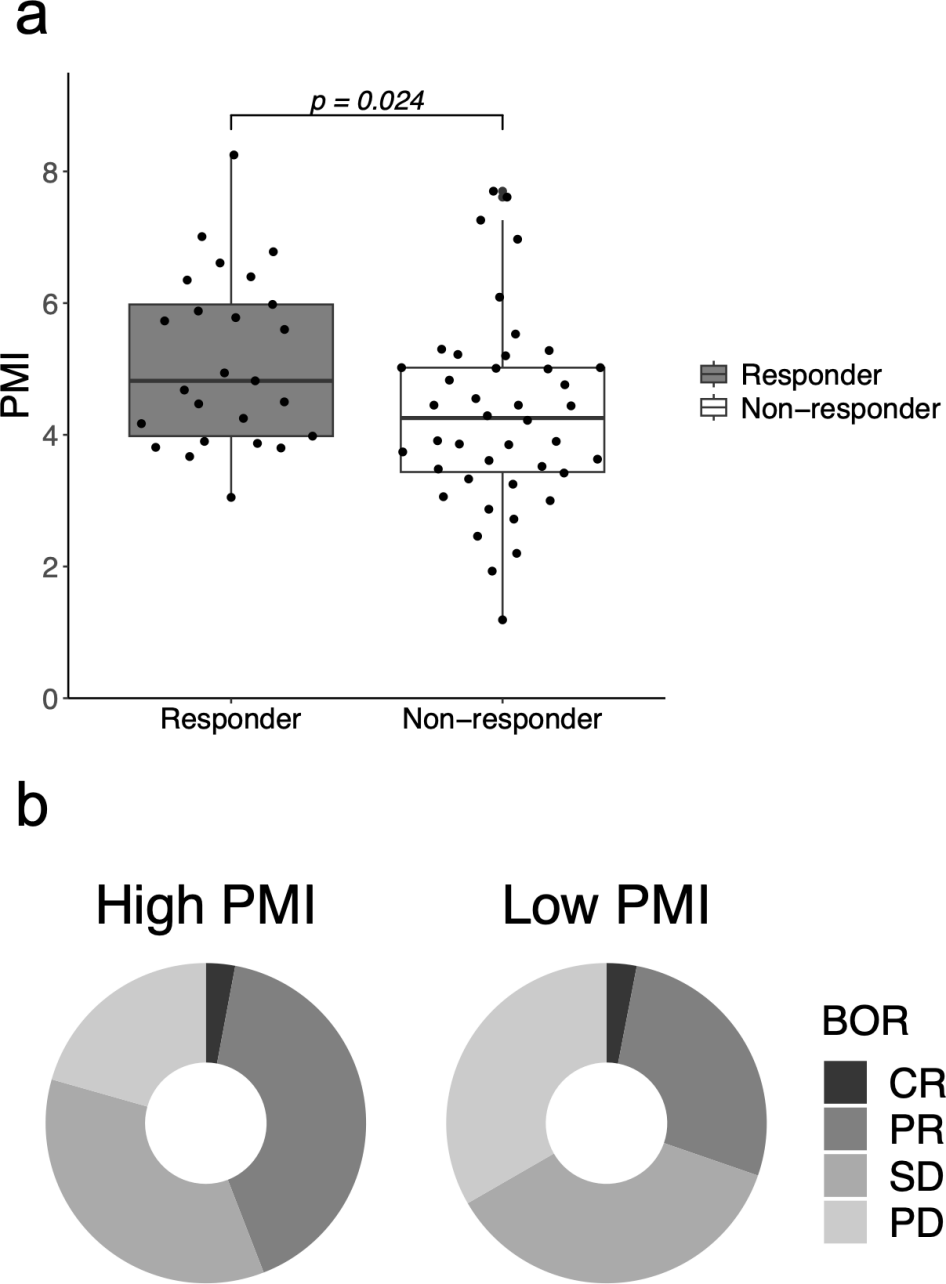
Clinical response to immune checkpoint inhibitors according to the psoas muscle index (PMI). **(A)** PMI in responders who showed complete response (CR) or partial response (PR) and in non-responders. **(B)** Best overall response (BOR) in the high- and low-PMI groups. BOR, best overall response; CR, complete response; PD, progressive disease; PMI, psoas muscle index; PR, partial response; SD, stable disease.

Overall, 25 patients (37%) responded to ICI therapy. PMI was associated with the overall response in the unadjusted logistic regression analysis (odds ratio [OR]: 1.52; 95% confidence interval [CI]: 1.04–2.22; *p* = 0.030), and the analysis adjusted for age (OR: 1.49; 95% CI: 1.01–2.21; *p* = 0.046). Sensitivity analysis with adjustment for sex and albumin level resulted in similar findings (**Supplementary Table 1**). In addition, PMI was independently associated with a better response to PD-L1 expression (adjusted OR for PD-L1 expression: 1.54; 95% CI: 1.04–2.27; *p* = 0.030). Responders (CR or PR) had a significantly higher mean PMI than that of non-responders (5.13 cm^2^/m^2^ vs. 4.31 cm^2^/m^2^; *p* = 0.024) (**Figure 2**). The high-PMI group had an ORR of 44%, whereas the low-PMI group had an ORR of 30% (OR: 0.55; 95% CI: 0.20–1.50). The disease control rates (DCR) were 79% and 67% in the high- and low-PMI groups, respectively (OR: 0.52; 95% CI: 0.17–1.56). However, ORR was not correlated with PNI (unadjusted OR: 1.07; 95% CI: 0.98-1.17; *p* = 0.144) or GNRI (unadjusted OR: 1.04; 95% CI: 0.99-1.08; *p* = 0.101).

### 3.4 Incidence of irAEs

**Figure 3:**
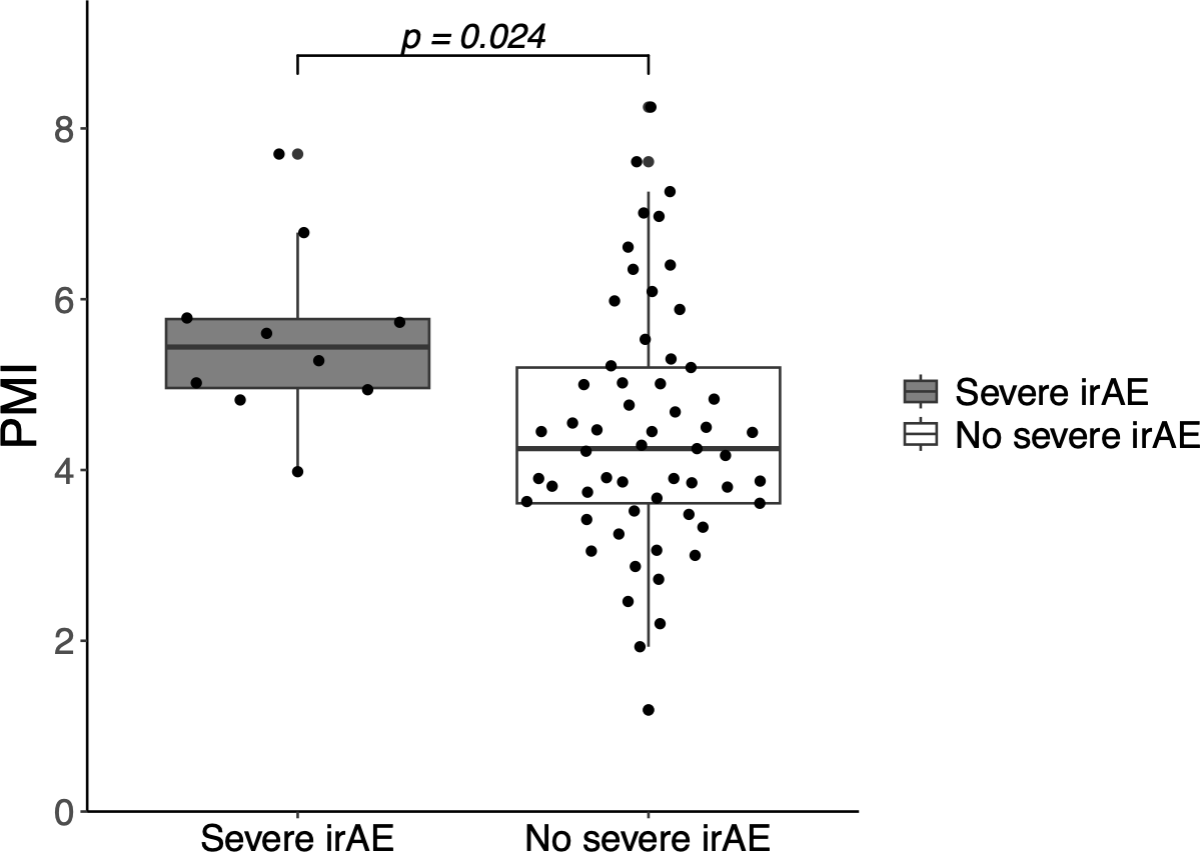
Incidence of severe immune-related adverse events (irAEs) according to the psoas muscle index (PSI). PMI, psoas muscle index; irAE, immune-related adverse event.

Nineteen patients (28%) experienced irAEs, and 10 (13%) experienced severe irAEs leading to ICI discontinuation. Logistic regression analysis showed a significant association between PMI and the incidence of severe irAEs (OR: 1.72; 95% CI: 1.05–2.80; *p* = 0.031), but did not show a significant association between PMI and all-grade irAEs (OR: 1.24; 95% CI: 0.853–1.80; *p* = 0.261). Multivariable logistic regression analysis also showed that PMI was independently associated with severe irAEs (**Table 2**). The mean PMI was significantly higher in patients with severe irAEs than in those without irAEs (5.56 cm^2^/m^2^ vs. 4.45 cm^2^/m^2^; *p* = 0.024) (**Figure 3**). The incidence of severe irAEs was 23.5% and 6% in the high- and low-PMI groups, respectively (OR: 0.21; 95% CI: 0.04–1.08). PNI was not associated with the incidence of severe irAEs (unadjusted OR: 1.12; 95% CI: 0.98-1.28; *p* = 0.098) nor was GNRI (unadjusted OR: 1.03; 95% CI: 0.98-1.09; *p* = 0.240).

**Table 2:** Multivariable logistic regression model of the association between PMI and the incidence of severe irAEs, adjusted for age.

Variables	Odds ratio (95% confidence interval)	*p*
PMI (cm^2^/m^2^)	1.71 (1.03–2.86)	0.039
Age (years)	1.00 (0.92–1.09)	0.996

irAE, immune-related adverse event; PMI, psoas muscle index.

### 3.5 PFS and OS

The median PFS and OS for all patients were 252 and 858 days, respectively. Patients in the high-PMI group had a longer median PFS and OS than those in the low-PMI group, but these differences were not statistically significant (PFS: 386 days vs. 186 days, *p* = 0.173; OS: 878 days vs. 551 days; *p* = 0.059) (**Supplementary Figure 1**). Cox regression analysis did not show a significant association between PMI and survival (hazard ratio [HR] for PFS: 0.95; 95% CI: 0.80–1.14; *p* = 0.594, HR for OS: 0.95; 95% CI: 0.78−1.16; *p* = 0.616). In contrast, nutritional indices such as PNI and GNRI were correlated with both PFS and OS. Higher PNI values were associated with longer PFS (HR: 0.95; 95% CI: 0.91-1.00; *p* = 0.030) and OS (HR: 0.93; 95% CI: 0.88-0.98; *p* = 0.008). Similarly, higher GNRI values showed a association with longer PFS (HR: 0.97; 95% CI: 0.95-1.00; *p* = 0.051) and OS (HR: 0.96; 95% CI: 0.93-0.99; *p* = 0.015). Multivariable analysis with adjustment for age resulted in similar findings (**Supplementary Table 2**).

### 3.6 IrAEs and clinical outcomes

Further analyses were performed to examine the association between the presence of irAEs and clinical outcomes. Multivariable logistic regression analysis showed that ORR in patients with irAEs was higher than in those without (63% vs. 27%; OR adjusted for age: 5.14; 95% CI: 1.61–16.4; *p* = 0.006). The log-rank test revealed that the presence of irAEs was strongly associated with both PFS (median PFS: 625 days with irAEs and 146 days without irAEs; *p* = 0.011) and OS (median OS: 1,030 days with irAEs and 523 days without irAEs; *p =* 0.031) (**Supplementary Figure 2**). Cox regression analysis also revealed that the presence of irAEs was independently associated with both PFS and OS (**Supplementary Table 3**).

## 4 Discussion

This study demonstrated a significant association between the ORR to ICIs and PMI. Notably, patients with a higher PMI had a higher incidence of irAEs, which are thought to be caused by ICI-induced immune cells. These findings indicate that PMI can serve not only as a prognostic indicator in patients with cancer but also as a predictor of their response to ICI therapy.

First, PMI was correlated with the ORR to ICIs, with responders having a significantly higher mean PMI than non-responders. Grouping patients based on the cutoff value of the median PMI showed higher ORR and DCR in the high-PMI group than in the low-PMI group. Furthermore, the mean PFS and OS in the high PMI group were double those in the low-PMI group, although these differences were not statistically significant. The lack of statistical significance is attributable to the small sample size. Recently, several parameters have been evaluated as biomarkers for predicting the response to ICI therapy. Of these, skeletal muscle mass is correlated with survival in patients with advanced NSCLC treated with nivolumab or a combination of ICIs and cytotoxic chemotherapy (5, 6). Additionally, PMI is correlated with the response to PD-1 inhibitors in patients with NSCLC (7). Similar associations have also been reported in patients receiving ICIs for gastric cancer and renal cell carcinoma (8–11). Two independent meta-analyses have shown that sarcopenia has a negative effect on the overall response and survival in patients treated with ICIs for various types of cancer, consistent with the results of this study.

Second, this study revealed a strong association between the PMI and the incidence of irAEs. This contrasts with previous reports on conventional cytotoxic chemotherapy, in which patients with sarcopenia tend to have more adverse events and higher discontinuation rates (1, 19–22). The difference in the effect of PMI on response to ICIs and cytotoxic chemotherapy can be attributed to fundamental differences in the mechanisms of these adverse events. Biologically, irAEs are inflammatory toxicities induced by overactivated T cells reinvigorated by ICIs (23). In a mouse model of ICI-induced myocarditis, clonally expanded cytotoxic CD8^+^ T cells infiltrated the myocardium and were necessary for the development of myocarditis (24). Similar findings have been reported for ICI-induced colitis. Single-cell RNA sequencing of colon biopsies from patients with ICI-induced colitis revealed an expansion of cytotoxic CD8^+^ T cells (25).

These findings suggest that the antitumor effects of ICIs and irAEs have a common underlying mechanism. In other words, the incidence of irAEs is a sign of sufficient activation of the host immune system by ICIs, and patients who experience irAEs can be deemed to be “responders” to ICIs. The presence of irAEs was strongly associated with both the clinical response of ICIs and survival in the present study. Several studies have had similar findings in NSCLC and in other types of cancer (26–29). Furthermore, severity and the number of irAEs were also associated with better survival in patients with NSCLC (30–32). This suggests that irAEs may not only reflect immune system activation but also act as an indicator of the degree of immune engagement, which could contribute to improved long-term outcomes.

The associations between PMI and both clinical response and irAEs suggest that PMI is not only a prognostic indicator but can also be used as a surrogate marker for patient immunocompetence, which predicts the clinical response to ICIs. Skeletal muscle cells secrete various cytokines called myokines, which communicate with other organs, such as the liver, pancreas, cardiovascular system, brain, and bones (33). Recently, skeletal muscle has been shown to regulate both innate and adaptive immune responses via surface molecules and myokines, and loss of skeletal muscle mass and sarcopenia may cause immune senescence (3, 4). Preclinical research has shown that skeletal muscle also interacts with the gut microbiota, which affect the tumor microenvironment via their metabolites (34–36). A clinical study of patients with lung cancer has shown that sarcopenia is associated with changes in the gut microbiota and their metabolites (37). These connections between skeletal muscle and the immune system may explain the greater potential utility of PMI for predicting the effectiveness of ICIs than for predicting response to conventional cytotoxic chemotherapy. In our study, PMI was independently associated with a better response to PD-L1 expression. Our findings suggest that PMI complements and enhances the prediction of clinical outcomes alongside PD-L1 expression, an established biomarker. While PD-L1 expression primarily focuses on tumor characteristics, this result further supports the idea that PMI reflects the patient’s overall immune status, which influences their response to ICIs. However, a recent meta-analysis, which found that sarcopenia had a negative effect on the clinical response to ICIs, was inconclusive regarding the relationship between sarcopenia and irAEs (38, 39). Considering that this lack of clarity is possibly due to the limited number of studies on irAEs, additional large studies are necessary to reach a conclusion.

Although PMI was significantly associated with both clinical response (ORR) and the incidence of severe irAEs, nutritional indices such as PNI and GNRI were not correlated with these factors but were linked to longer PFS and OS. This suggests that PMI is more closely related to immune response and may serve as a marker for immunocompetence, influencing both the clinical response to ICIs and the occurrence of irAEs. In contrast, PNI and GNRI appear to function more as prognostic scores, reflecting long-term survival outcomes rather than immediate treatment response. This distinction underscores the multifaceted relationship between muscle mass, nutrition, and immune status in patients treated with immune checkpoint inhibitors. Further research is needed to elucidate the mechanisms by which these indices influence immunotherapy outcomes.

Importantly, one strength of this study is that it focuses exclusively on patients treated with ICI monotherapy. By excluding combination therapies, we could better isolate the impact of ICI therapy alone, without the confounding effects of chemotherapy, which can also influence both objective response rates (ORR) and survival outcomes. However, this study has several limitations. First, it was conducted at a single institution with a relatively small sample size, which may limit the statistical power and generalizability of our findings. Additionally, the homogeneity of our cohort, composed exclusively of Asian patients, may restrict the applicability of our results to more diverse populations. The study also included relatively few female patients, which may limit the generalizability of the findings with regard to sex-based differences in sarcopenia or immune response. Furthermore, the use of previously reported cut-off values for PMI, which were established in healthy individuals without cancer, may not fully reflect the optimal thresholds for sarcopenia in cancer patients. These reference values (3.92 cm²/m² for female patients and 6.36 cm²/m² for male patients) led to a high proportion of patients in our cohort being classified as sarcopenic (87% of female patients and 83% of male patients), potentially limiting the generalizability of these thresholds to cancer populations. The optimal cut-off points for sarcopenia, particularly in the context of cancer treatment, remain an area of ongoing research. Moreover, as this was a retrospective study, we were unable to perform immune profiling, such as evaluating effector and regulatory T cells, myeloid-derived suppressor cells, natural killer cells, or monocyte activity, which may have provided deeper insights into the relationship between skeletal muscle mass and immune competence. Future prospective studies incorporating detailed immune profiling will be essential to elucidate the mechanisms underlying the observed associations between PMI and clinical outcomes in patients treated with ICIs. Additionally, the question of whether interventions to improve the sarcopenic status of patients with cancer can improve ICI response and overcome resistance to ICIs remains unanswered. Several interventions such as nutritional support, physical exercise, and anamorelin have been investigated, but none of these interventions have demonstrated clear clinical benefits on the survival of patients with cancer (1, 40). An ongoing trial including patients with melanoma receiving ICI therapy, with the intervention group undergoing diet modulation and regular exercise, is assessing the effect of exercise on response to ICI therapy (NCT04866810). Finally, the retrospective nature of this study means that we cannot infer causality, and the design inherently introduces selection bias, as well as the potential for residual confounders not accounted for in the analysis. Therefore, prospective clinical trials with well-defined interventions to increase patients’ PMI are required to provide conclusive evidence on the relationship between PMI and response to ICI therapy.

In conclusion, this study demonstrated a significant association between PMI and response to ICI therapy in patients with NSCLC. The incidence of irAEs, which are thought to be a sign of immune cell reinvigoration by ICIs, was also associated with PMI. These findings suggest that PMI may serve as a prognostic indicator and a biomarker of immunocompetence that can predict response to ICI therapy.

## Data Availability

The raw data supporting the conclusions of this article will be made available by the authors, without undue reservation.
