# Supplementary material for: Evaluation of patient immunocompetence for immune checkpoint inhibitor therapy using the psoas muscle index: a retrospective cohort study
Source: Front Oncol. 2025 Feb 6;15:1499650. doi: 10.3389/fonc.2025.1499650 (PMC11839410; doi:10.3389/fonc.2025.1499650)
Supplement: Supplementary file 1 [file DataSheet1.docx]

Supplementary Material

# Supplementary Figures and Tables

## **Supplementary** Figures


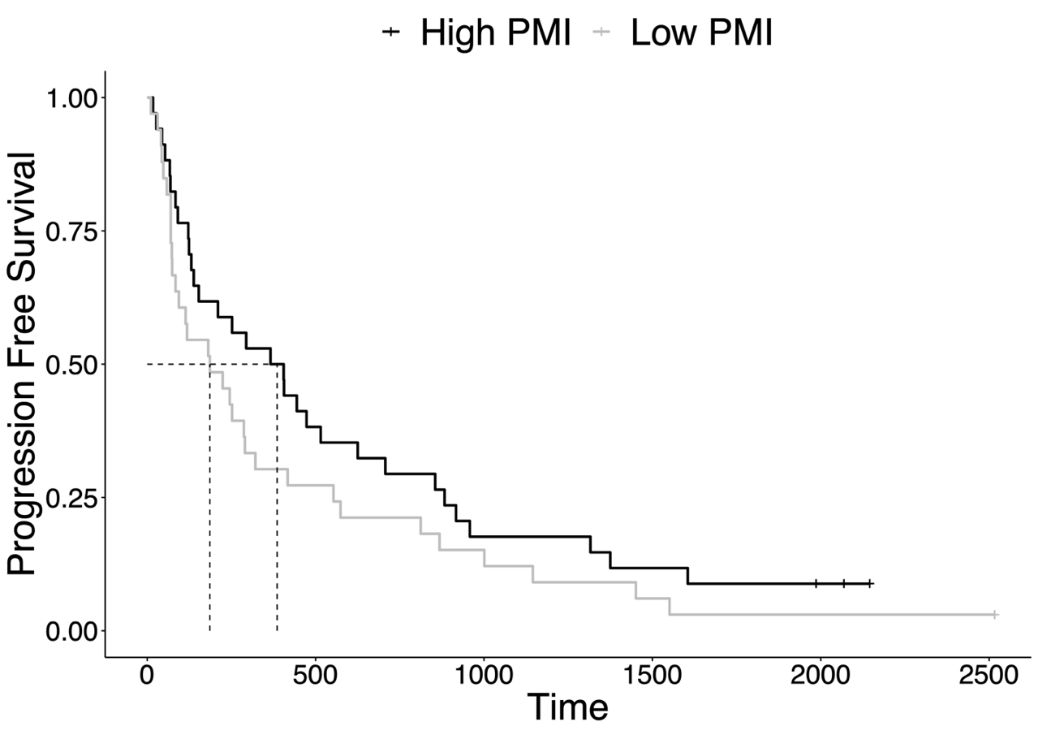


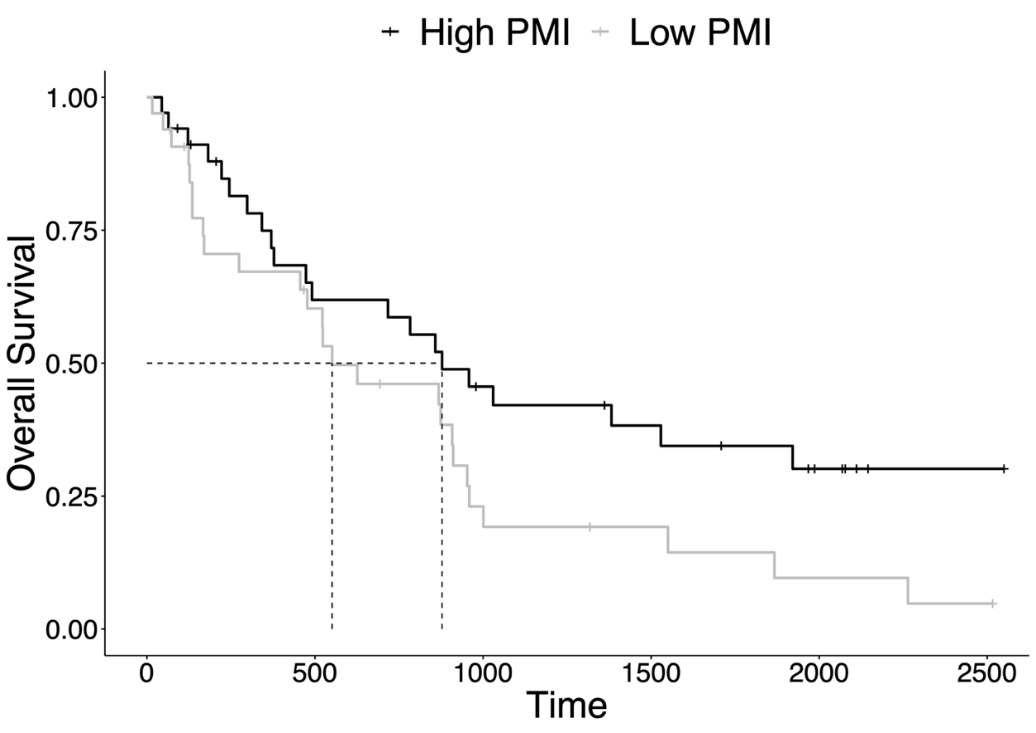


**Supplementary Figure 1.** Kaplan–Meier plots showing progression-free survival (PFS) and overall survival (OS) according to psoas muscle index (PMI) status. (A) PFS; (B) OS.


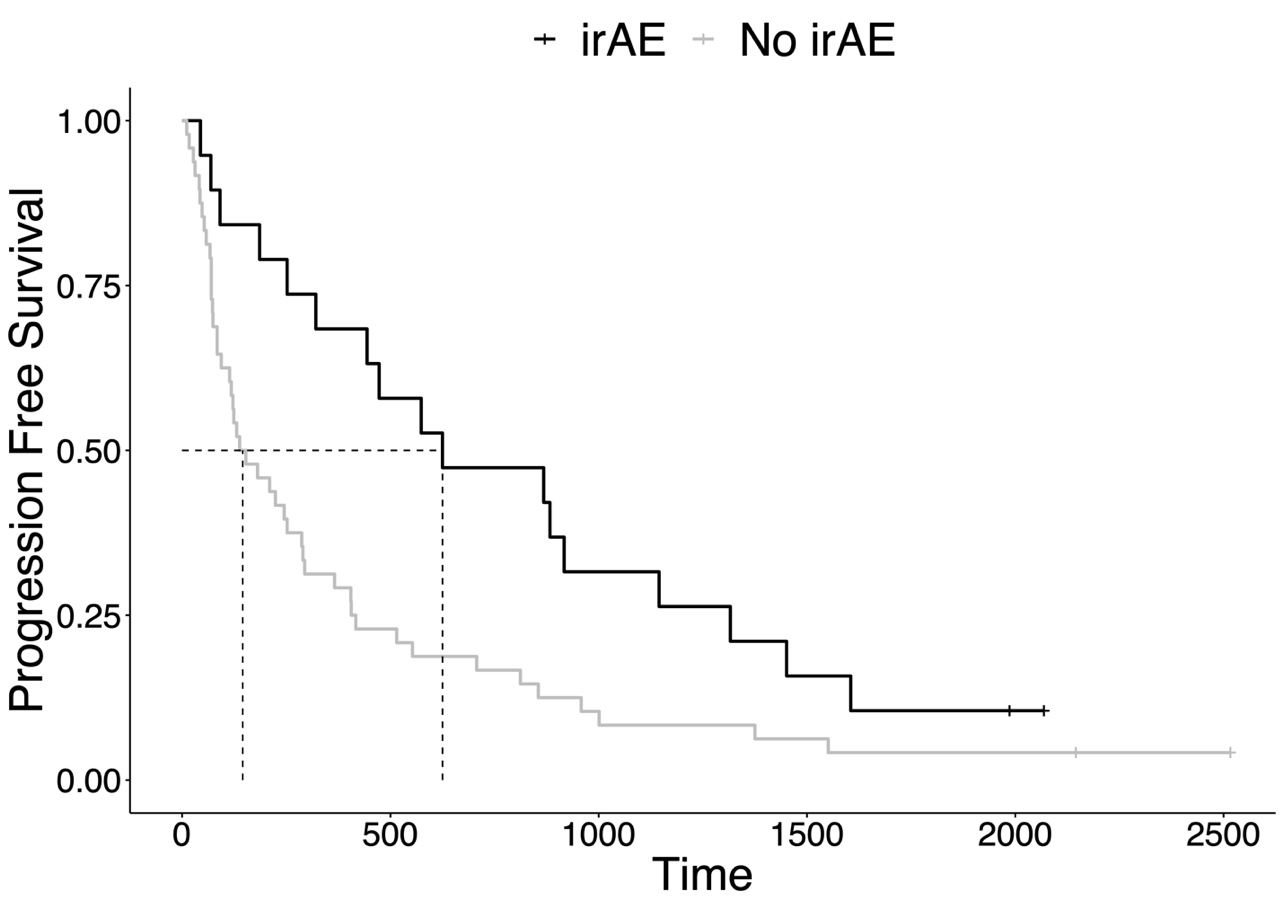

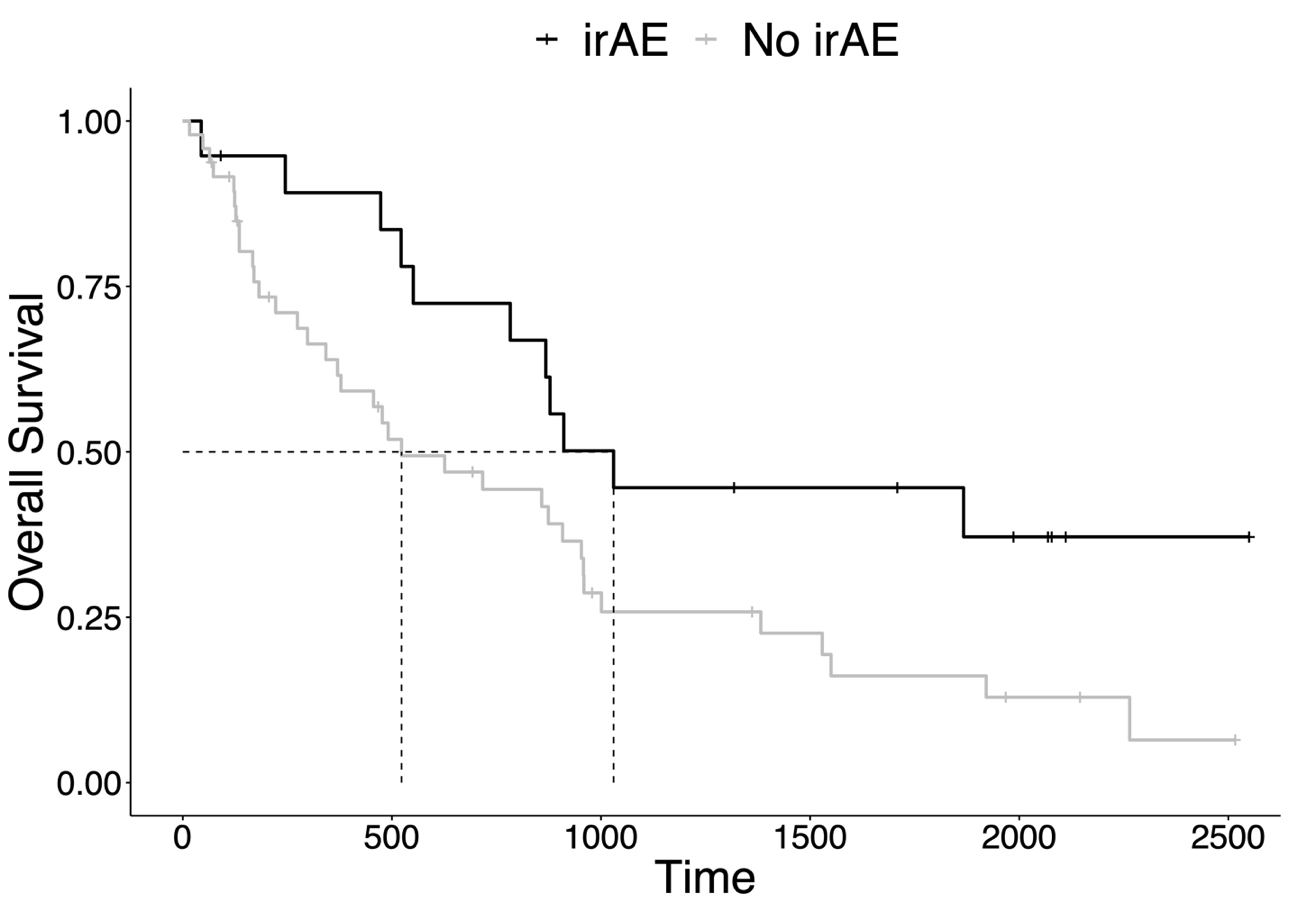


**Supplementary Figure 2.** Kaplan–Meier plots showing progression-free survival (PFS) and overall survival (OS) according to the presence of immune-related adverse events (irAEs). (A) PFS (B) OS.

## **Supplementary** Tables

**Supplementary Table 1. Multivariable logistic regression models of the overall response to immune checkpoint inhibitor therapy**

1. **Odds ratio adjusted for sex**

| Variables | Odds ratio (95% CI) | *p* |
| --- | --- | --- |
| PMI (cm^2^/m^2^) | 1.54 (1.00–2.36) | 0.048 |
| Sex (Male) | 0.93 (0.22–3.99) | 0.919 |

1. **Odds ratio adjusted for serum albumin level**

| Variables | Odds ratio (95% CI) | *p* |
| --- | --- | --- |
| PMI (cm^2^/m^2^) | 1.49 (1.01–2.19) | 0.046 |
| Serum albumin level (g/dL) | 1.94 (0.69–5.45) | 0.210 |

PMI, psoas muscle index; CI, confidence interval

**Supplementary Table 2. Cox regression models for progression-free survival (PFS) and overall survival (OS), adjusted for age**

| Variables | PFS | |  | OS | |
| --- | --- | --- | --- | --- | --- |
|  | Hazard ratio (95% CI) | *p* |  | Hazard ratio (95% CI) | *p* |
| PNI | 0.95 (0.91–1.00) | 0.030 |  | 0.93 (0.88–0.98) | 0.006 |
| GNRI | 0.97 (0.95–1.00) | 0.053 |  | 0.96 (0.93–0.99) | 0.013 |

CI, confidence interval; GNRI, Geriatric Nutritional Risk Index; OS, overall survival; PFS, progression-free survival; PNI, Prognostic Nutritional Index

**Supplementary Table 3. Cox regression of factors associated with progression-free survival (PFS) and overall survival (OS)**

| Variables | PFS | |  | OS | |
| --- | --- | --- | --- | --- | --- |
|  | Hazard ratio (95% CI) | *p* |  | Hazard ratio (95% CI) | *p* |
| irAE | 0.34 (0.17–0.69) | 0.003 |  | 0.44 (0.20–0.97) | 0.043 |
| Age | 0.99 (0.96–1.03) | 0.622 |  | 0.99 (0.95–1.03) | 0.490 |
| Sex (male) | 0.63 (0.29–1.37) | 0.239 |  | 1.48 (0.60–3.66) | 0.391 |
| NLR | 1.07 (0.95–1.19) | 0.232 |  | 1.15 (1.02–1.29) | 0.022 |

CI, confidence interval; irAE, immune-related adverse event; NLR, neutrophil-to-lymphocyte ratio; OS, overall survival; PFS, progression-free survival
